# Neural Oscillations: Sustained Rhythms or Transient Burst-Events?

**DOI:** 10.1016/j.tins.2018.04.004

**Published:** 2018-07

**Authors:** Freek van Ede, Andrew J. Quinn, Mark W. Woolrich, Anna C. Nobre

**Affiliations:** 1 Oxford Centre for Human Brain Activity, Wellcome Centre for Integrative Neuroimaging, Department of Psychiatry, University of Oxford, Oxford, UK; 2 Department of Experimental Psychology, University of Oxford, Oxford, UK

**Keywords:** local field potential, electroencephalography, magnetoencephalography, beta oscillations, signal interpretation, frequency analysis

## Abstract

Frequency-specific patterns of neural activity are increasingly interpreted as transient bursts of isolated events rather than as rhythmically sustained oscillations. This has potentially far-reaching implications for theories of how such oscillations originate and how they shape neural computations. As this debate unfolds, we explore alternative interpretations and ask how best to distinguish between them.

The recasting of neural oscillations as burst-events is gaining momentum, and is already inspiring the quantification of novel neural parameters such as burst-rate. The ‘bursting’ interpretation comes with far-reaching implications, but of course its significance hinges on it being an accurate reflection of the physiological measurements in each particular case. This Forum article aims not to argue for, or against, bursts, but instead aims to help to guide this incipient debate onto productive tracks. To do so, we clarify what burst versus sustained oscillation-based interpretations of frequency-specific neural activity precisely entail, and we explore methodological approaches that may be well suited for arbitrating between these interpretations. In the process, we illustrate how the two scenarios can be easily confused when it comes to realistic (noise-containing) neural data, and we raise the possibility of a novel ‘hybrid’ scenario.

This Forum article comments specifically on recent studies of beta (∼15–30 Hz) and gamma (∼40–100 Hz) oscillations in humans and non-human primates 1, 2, 3, 4, 5. Although the points raised may be particularly applicable to these phenomena, they could possibly be relevant for other frequency-specific neural phenomena as well. In the following we use the term ‘frequency-specific patterns of neural activity’ to refer to a larger set of phenomena that are typically labeled ‘neural oscillations’. We emphasize that the answer to our title question may well turn out to be different for different phenomena (e.g., slow wave vs spindle activity during sleep), or even the same apparent phenomenon in different contexts (e.g., sensorimotor beta activity during rest vs tonic contraction). It will thus be vital for this debate to be held for each frequency-specific pattern of neural activity separately, and the considerations below, when applied to each specific case, are not necessarily expected to converge into a single, overarching picture that is equally applicable to all neural systems, species, and frequency bands. Indeed, it is likely that both burst-events and sustained oscillations coexist in the brain and, in some cases, these may even occupy similar frequency ranges.

## Origin and Relevance of the Debate

Frequency-specific patterns of neural activity, as observed in local field potentials (LFPs) or magneto/electroencephalography (M/EEG), have traditionally been thought of as sustained rhythmic fluctuations in the excitability of the underlying neural populations. Such ‘oscillations’ have come to the fore of the scientific quest for the mechanisms by which the brain flexibly codes and routes information [6]. Recently, however, several studies in humans and non-human primates have questioned the very nature of some of these oscillations – namely, their regular recurrence or ‘rhythmicity’ 1, 2, 3, 4, 5. By evaluating time–frequency data at the level of single trials it has been suggested that neural activity – particularly in the beta- and gamma-frequency ranges – may come in small packets, or ‘bursts’, that last only one or very few cycles. It is only through the averaging (e.g., over trials) of time-varying bursts that these phenomena (and their task-related modulations) appear to be sustained. Thus, instead of conceiving primate beta- and gamma-band activity as sustained oscillations, these recent studies invite us to reconsider such activity as transient, isolated burst-events which can be described not only by their frequency and amplitude but also by their rate, timing, duration, and shape.

Embracing this reinterpretation of oscillations as burst-events has far-reaching consequences. Conceptually, it challenges models of how such ongoing oscillations serve to route information flexibly through neural networks to enable, for instance, sustained selective attention [6] and working memory [2]. While bursts may still enable transient communication channels between neural populations, it is more difficult to envisage how bursts may enable longer-lasting communication channels to facilitate, for example, working memory retention. It also challenges models proposing that such oscillations are generated through ongoing recurrent excitation and inhibition (e.g., [7]). In practice, the burst-event picture prompts us to conceive of new ways to quantify these events and to chart their parameters. It may further impact on clinical models and therapeutic interventions for cases of aberrant ‘oscillations’ – such as beta oscillations in Parkinson’s disease [5].

In the remainder we clarify what the possible alternative interpretations of frequency-specific neural activity precisely entail, and we outline some guiding principles on how one may best arbitrate among them.

## Conceptual Alternatives

**Figure 1:**
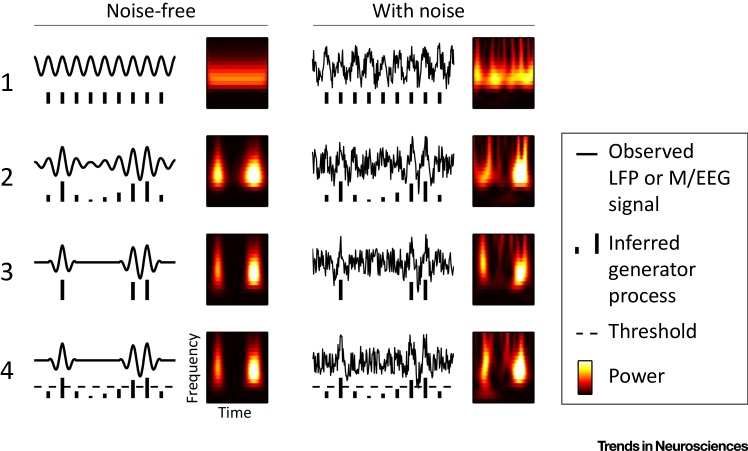
Four Alternative Conceptual Scenarios. (1) Rhythmically sustained oscillation without amplitude dynamics. (2) Rhythmically sustained oscillation with amplitude dynamics. (3) Burst-events with no underlying rhythmicity. (4) A hybrid scenario in which the consequences of rhythmically sustained generator processes depend on a certain threshold being crossed. Time–frequency maps were estimated using a wavelet analysis with 3 cycles per wavelet. Time and frequency units are arbitrary. Abbreviations: LPF, local field potential; M/EEG, magneto/electroencephalography.

We consider four alternative interpretations of frequency-specific activity patterns in LFP or M/EEG measurements (Figure 1). For each scenario, the schematic shows a hypothetical signal (left columns), and the same signal with noise (right columns), mimicking data as typically observed in LFP and M/EEG measurements. The time-domain signals are shown alongside their signal generators (below), which in real situations most often remain unavailable and are therefore inferred. For simplicity, we consider these generators as ‘pulses’, which determine the amplitude and timing of the observed activity, but whose precise physiological origins are beyond the remit of this article ([7] for discussion) and may differ between scenarios. For completeness, we also show the associated time–frequency map of power next to each time-domain signal.

Scenario 1 shows the simplest model in which the observed oscillatory signal is generated by rhythmic pulses (i.e., a pacemaker) with constant amplitude. Next, by adding time-varying modulation in the strengths of these generating pulses, we arrive at a model that resembles more closely the dynamics of brain activity as often measured empirically (scenario 2). The strength of these pulses may be dependent, for example, on the summation of inputs or on global excitatory–inhibitory balance. We consider that this the ‘standard model’ for interpreting frequency-specific brain activity: while the amount of spectral energy may vary, for example, with physiological state and task context, the underlying pulsing shows sustained rhythmicity (although this may not always be apparent from the corresponding time–frequency maps; Figure 1). By contrast, in the emerging ‘burst’ view (scenario 3), the underlying pulses no longer occur rhythmically, but occur stochastically instead. While individual pulses may still generate physiological responses whose characteristic shapes are best captured in a particular frequency range (e.g., beta-bursts), the generator pulses themselves are no longer rhythmic. Finally, we raise the possibility of a novel (to our knowledge) ‘hybrid’ account (scenario 4) in which the generator pulses are rhythmic, but where a threshold (dashed line, Figure 1) determines whether any given pulse will result in a measurable burst-event. When single pulses cross the threshold, isolated bursts result (left pulse, scenario 4), whereas when multiple successive pulses cross the threshold, multi-cycle oscillations result (right pulses, scenario 4). Although purely hypothetical, this scenario could possibly provide a single substrate to both types of phenomena in observed measurements.

The behavioral state, it is worth mentioning, is likely to play a major role in determining parameters of frequency-specific activity. The duration over which sustained oscillations or bursts occur is likely to be linked to their utility in guiding current behavior. In scenario 4, for example, the period over which a threshold is crossed may depend on behavioral demands, yielding transient bursts for fast computations but sustained oscillations for more prolonged computations.

## Means of Arbitration

When individual trials differ in the precise timing of their amplitude dynamics or bursts, trial-averaging may yield patterns that appear sustained, even for scenarios 2–4. We set this single-trial versus average issue aside here (2, 8, 9 for discussion), and ask how single-trial scenarios 2 and 3 themselves can be distinguished. In other words, we do not question that brain activity (before averaging) will vary over time, but instead ask what form this variability takes – isolated burst-events or dynamic amplitude variations of sustained oscillations. This is particularly non-trivial when only LFP or M/EEG data are available. In the presence of noise (Figure 1, right column), periods of sustained, but low-amplitude, oscillations (scenario 2) may be obscured, and spurious amplitude fluctuations may be introduced. Moreover, periods of low-amplitude oscillations may remain hidden in time–frequency maps of power, even when dealing with clean signals (scenario 2, Figure 1).

One approach is to use amplitude-thresholding to identify putative bursts. While thresholding opens the door to quantifying novel burst parameters 1, 2, 3, 4, 5, which may vary with task and performance in interesting ways 1, 2, 4, we stress that the interpretation of such parameters is only meaningful if the burst scenario is indeed valid – of course, burst statistics may still provide a sensitive proxy of amplitude dynamics in scenario 2, and vice versa for amplitude statistics in scenario 3. It is thus vital to establish ways to arbitrate among the scenarios.

An important feature that sets the burst scenario apart is the lack of continuous phase-progression between successive timepoints – and therefore the ability to predict the future phase of the signal – at least beyond the borders of individual bursts. Measures that capture the rhythmic regularity of such phase-preservation [10] could thus be key in advancing this debate. Although such measures are currently not mainstream, one relevant previous study investigated sensorimotor beta activity in MEG data, and argued that this activity can be well characterized by such phase-preservation [10], at least for the temporal intervals over which phase-preservation was considered in that study. Increasingly popular accounts representing the same type of sensorimotor beta activity as transient bursts 1, 3, 4 predict that the temporal extent of its phase-preservation will be bound to the duration of individual bursts. In future endeavors it will thus be informative to systematically map out the temporal extent of phase-preservation – and this will be relevant not only for sensorimotor beta activity but also for all other frequency-specific phenomena at stake. It will also be important to directly compare phase-preservation with thresholding approaches to achieve a better grasp of their scope, utility, and limitations. One noteworthy feature of phase-preservation measures is that they can be aggregated across time and trials such that estimates will become increasingly accurate with more data.

As our schematic hopefully makes clear, advancing this debate will require data with high signal-to-noise ratio (SNR). Complementing this, we anticipate valuable contributions from methods that move beyond the predefined temporal windowing and linearity constraints of conventional Fourier-based analyses,and that do not easily adapt to the timescales of the dynamics. Two prime candidates are empirical mode decomposition [11], which also brings increased temporal and spectral resolution, and hidden Markov modeling, which provides a natural description of time-series as a sequence of states that can help to boost the effective SNR by pooling over different state-visits (e.g., in different trials). These approaches offer useful alternative descriptions of the time-varying nature of brain activity before averaging – including specific information about the timing and duration (‘lifetimes’) of states, which can then be related to behavior [12]. The connectivity profiles associated with such states may further enrich the debate by inviting consideration of frequency-specific phenomena related to functional coupling between regions, thereby unlocking a next set of fundamental questions relating to inter-areal coupling.
